# Alpha-pinene ameliorate behavioral deficit induced by early postnatal hypoxia in the rat: study the inflammatory mechanism

**DOI:** 10.1038/s41598-024-56756-1

**Published:** 2024-03-17

**Authors:** Sharareh Bakhtazad, Zohreh Ghotbeddin, Mohammad Reza Tabandeh, Kaveh Rahimi

**Affiliations:** 1 Department of Basic Sciences, Faculty of Veterinary Medicine, Shahid Chamran University of Ahvaz, Ahvaz, Iran; 2 Stem Cell and Transgenic Technology Research Center, Shahid Chamran University of Ahvaz, Ahvaz, Iran; 3 Department of Biochemistry and Molecular Biology, Faculty of Veterinary Medicine, Shahid Chamran University of Ahvaz, Ahvaz, Iran

**Keywords:** Alpha-pinene, Behavioral responses, Hypoxia, Inflammation, Rat, Biological techniques, Neuroscience, Physiology

## Abstract

Neonatal hypoxia has a negative impact on the developing brain during the sensitive period. Inflammation plays a key role in the physiological response to hypoxic stress. Considering the anti-inflammatory properties of alpha-pinene, which has received a lot of attention in recent years, in this research we focused on the impact of alpha-pinene on the behavioral responses and proinflammatory factors in rats subjected to the neonatal hypoxia. This study involved Wistar rats (7-day-old) that were divided into six experimental groups, including a control group, groups receiving different doses of alpha-pinene (5 and 10 mg/kg), a hypoxia group receiving 7% O_2_ and 93% N_2_, 90 min duration for 7 days, and groups receiving alpha-pinene 30 min before hypoxia. All injections were done intraperitoneally. The rats were evaluated for proinflammatory factors 24 h after exposure to hypoxia (PND14) and at the end of the behavioral test (PND54). The results showed that hypoxia led to decreased motor activity, coordination, and memory, as well as increased inflammation. However, the rats that received alpha-pinene showed improved behavioral responses and reduced inflammation compared to the hypoxia group (all cases *p* < 0.05). This suggests that alpha-pinene may have a protective effect via anti-inflammatory properties against the negative impacts of hypoxia on the developing brain.

## Introduction

Hypoxia may occur during pregnancy, childbirth, or in the postpartum period^1^. Hypoxia, or lack of oxygen, can lead to behavioral impairments in animals, affecting motor coordination, sensory integration, learning, and cognition. Hypoxia may also lead to anxiety-like behavior, hyperactivity, and sensory-motor development delays in animals^2^. The hypoxia model in rodents is a suitable model for the generalization of hypoxia in humans, which was first done by Rice et al. The application of hypoxia during the period of 6 to 12 days after birth, which is a critical period of synaptic maturation, can be associated with different disorders^3^.

The model of repeated neonatal hypoxia without ischemia was used in the present work. The choice of the model and the specific period of PND7-PND13 have been based on previous research indicating that this time frame is critical for brain development and vulnerability to hypoxic insults. The model can replicate the effects of chronic or repeated hypoxia, which can occur in certain clinical conditions such as chronic lung disease in premature infants or sleep apnea. The physiological and pathological processes that this model aims to replicate may include the effects of repeated hypoxia on brain development, neuronal damage, and potential long-term neurological deficits. The model may be relevant to studying conditions such as perinatal asphyxia, and other neonatal brain injuries associated with hypoxia.

Inflammation is the main component of brain injury in hypoxia during infancy^4^ and inhibiting pro-inflammatory factors can be effective and protect the nervous system from hypoxia-related brain injury^5^. Microglia, resident macrophages of the CNS, are among the first cells to be activated after HI^6,7^. Activated microglia migrate to damaged areas^8^ and produce inflammatory cytokines (IL-6, IL-1β, TNF-α, and NF-kB), glutamate, nitric oxide and free radicals^9^. Neuroinflammation has been linked to a variety of behavioral problems, including depression, anxiety, and cognitive impairment. When the brain is inflamed, it can lead to changes in neurotransmitter levels, disrupted synaptic function, and impaired neurogenesis, all of which can contribute to behavioral issues. Inflammation in the brain can also affect the function of the hypothalamic–pituitary–adrenal (HPA) axis, which plays a key role in regulating stress responses. Dysregulation of the HPA axis can lead to increased levels of stress hormones such as cortisol, which have been associated with mood disorders and anxiety.

Furthermore, inflammation can also impact the blood–brain barrier, allowing harmful substances to enter the brain and potentially exacerbate behavioral problem^10^.

Overall, neuroinflammation has been implicated in a range of behavioral problems, and reducing inflammation in the brain may be an important strategy for addressing these issues. This highlights the importance of research into anti-inflammatory compounds and therapeutic interventions for protecting the brain from damage and preserving healthy brain function^2,11^.

In recent years, researchers have been focusing on the potential benefits of alpha-pinene on the nervous system. Alpha-pinene is a polyphenolic compound found in various conifer plants, and studies have shown its anti-inflammatory and antioxidant effects. It has also been found to have neuroprotective properties, improving inflammatory cytokine levels in nervous system diseases. Evidence suggests that alpha-pinene may have positive effects on memory and learning impairment, making it potentially useful in managing dementia^12^. These studies have suggested the beneficial therapeutic potential of alpha-pinene and similar monoterpenes by improving the level of inflammatory cytokines in the nervous system diseases^13^. Evidence shows that alpha-pinene has positive effect on scopolamine-induced memory and learning impairment, and it has been suggested that alpha-pinene can be useful in the management of dementia, and memory and learning impairment with its neuroprotective potential^14^. A study by Proverde et al. investigated the effects of alpha-pinene on neurobehavioral disorder, oxidative damage, and inflammatory response following ischemic stroke in rats. The results showed that alpha-pinene significantly reduced cerebral edema and infarct size, improved neurobehavioral function, and restored antioxidant enzyme activity to normal levels while decreasing levels of inflammatory factors in the brain^15^. Overall, alpha-pinene shows promise in protecting the nervous system from inflammation and oxidative damage, and further research is needed to fully understand its potential benefits in managing hypoxia-related behavioral deficits. So, the aim of this study is to investigate the effect of alpha-pinene during neonatal hypoxia on behavior responses as well as the level of pro-inflammatory factors including interleukin-1β (IL-1β) and tumor necrosis factor α (TNF-α) in the rat brain.

## Meterials and methods

### Animals

The current study was approved by the Institutional Ethics Committee of the Shahid Chamran University of Ahvaz (EE/1400.2.24.42815/Scu.ac.ir) and was conducted to the ARRIVE guidelines. All methods were performed under the relevant guidelines and regulations.

To reduce stress, rats were transferred from the animal house to the laboratory one week before the experiment, gently handled every day and all experiments were done at minimum noises and vibrations.

Neonatal Wistar rats were kept in a standard condition, it was a clean and safe environment with appropriate bedding, food, water, and temperature control. 12 pups were assigned to each group and each litter divided into groups. The pups were weaned at PND22. Then, they were separated from their mother and kept in a fully controlled standard facility and had free access to water and food.

**Figure 1:**
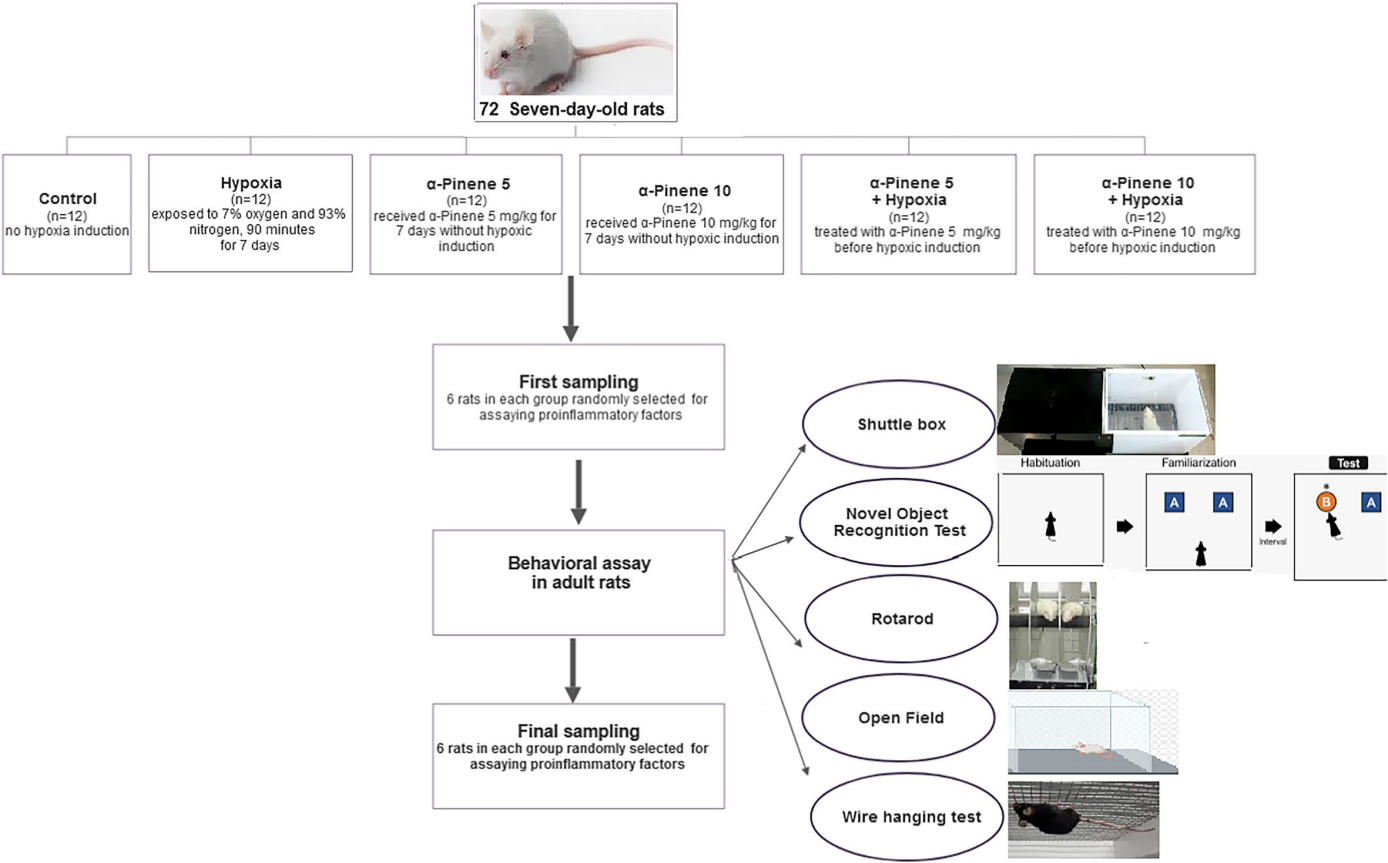
Schematic diagram of the test protocol.

All adult Wistar rats were placed in cages under standard laboratory conditions including 23–25 °C, 12 h light/dark, and ad libitum feeding. The rats were divided into 6 groups (with 6 rats in each group) as follows: (1) control, (2) hypoxia (were exposed to daily 90-min hypoxia during 7 days), (3) alpha-pinene pretreated with 5 mg/kg for 7 days without hypoxia induction, (4) alpha-pinene pretreated with 10 mg/kg for 7 days without hypoxia induction, and (5) alpha-pinene pretreated with 5 mg/kg for 7 days with hypoxia induction, and (6) alpha-pinene pretreated with 10 mg/kg for 7 days with hypoxia induction. The normoxic pups (groups 1, 3, 4) were exposed to the same experimental manipulations without hypoxia. The rats of control and hypoxia groups received daily vehicle injections. Alpha-pinene dissolved in a suitable solvent; corn oil and all injections were done intraperitoneally (Fig. 1).

### Hypoxia induction

The hypoxia box was a glass chamber, which had an air outlet and inlet valves. An oxygen and a nitrogen capsule were connected to the chamber. Temperature was maintained at 37.0 °C and relative humidity was maintained at 40–50 per cent.

The method to create the hypoxia model was that; 7-day-old rats were placed in the box and the air inlet and outlet valves were kept open for five minutes. after adjusting the entry of 7% oxygen and 93% nitrogen into the hypoxia chamber and checking the oxygen intensity with oximeter, both air inlet and outlet valves were closed and the rats were exposed to the air containing 7% oxygen and 93% nitrogen for 90 min. Temperature, lighting, and humidity were maintained at standard.

Following hypoxia exposure, the rat pups were returned to their nest and their mothers until weaning.

### Experimental assessment

**Figure 2:**
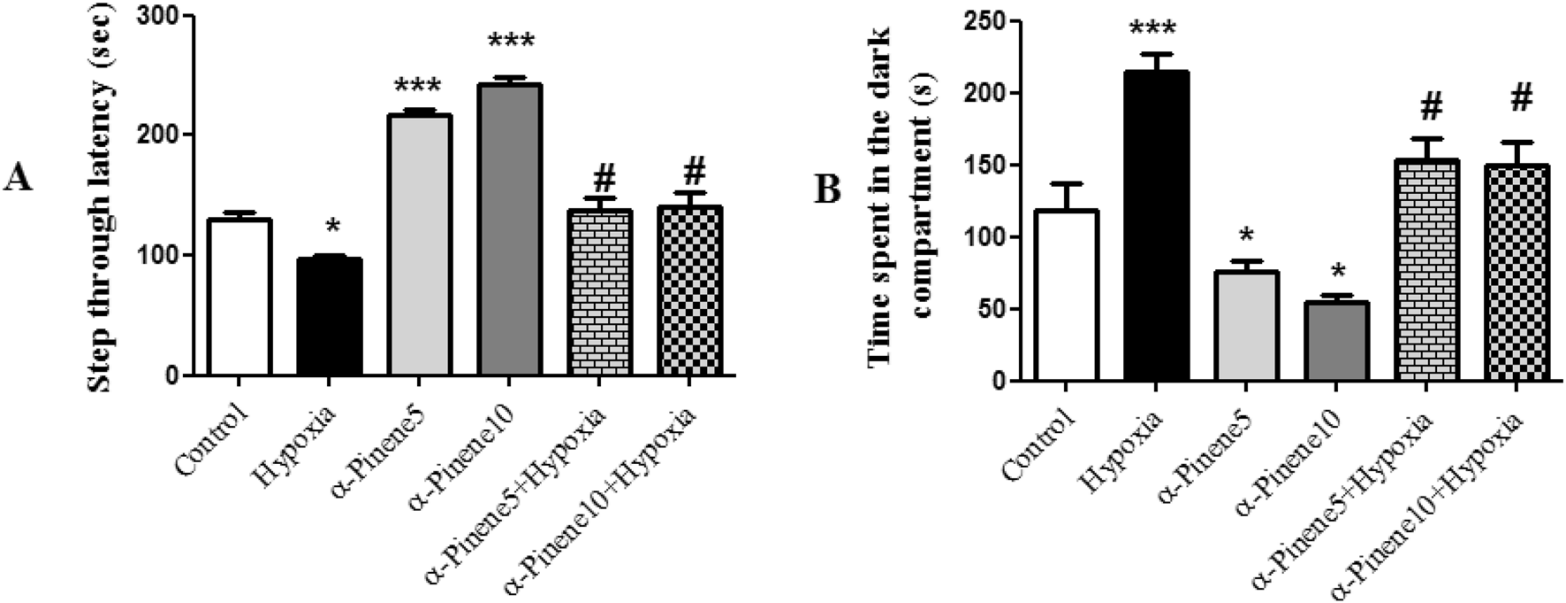
Learning deficits in the shuttle box test in the Hypoxia group. (**A**) The latency time of entrance to the dark compartment and (**B**) time spent in the dark compartment. Values represent the mean ± SEM, *** *p* < 0.001 and * *p* < 0.05 shows the difference between experimental groups versus Control group. # *p* < 0.05 indicates a significant difference between the hypoxia groups treated with alpha-pinenes and the hypoxia group (n = 6).

At PND14, 6 rats from each group were randomly selected to evaluate inflammatory cytokines. Other rats were kept until puberty for assaying both inflammatory cytokines and behavioral tests (Figs. 1 and 2). After behavioral tests, to assay pro-inflammatory cytokines, animals anesthetized with ketamine and xylazine mixture. Anesthetization of the animals was done by intravenously (IV) injection of ketamine and xylazine to induce sedation and analgesia. The dose and duration of this method depend on the weight and tolerance of the animals. For example, 80–120 mg/kg ketamine IV + 5 – 10 mg/kg xylazine IV can provide sedation for 30–45 min^16^.

### Assessment of pro-inflammatory cytokine

Pro-inflammatory cytokines were measured in rats of different ages: at the postnatal day 14 (PND14; 24 h after the last hypoxia session) and at PND54 (after behavior tests). They were assessed in the hippocampus by applying IL-1β and TNF-α rat sandwich enzyme-linked immunosorbent assay (ELISA) kit (Kiazist, Iran). The samples were homogenized within 500 µl of lysis buffer and centrifuged at 11,000 g for 15 min at 4 °C. The supernatants were diluted with diluent buffer and added to each well of the ELISA kits. According to the manufacturer’s protocol, the ELISA procedure was done. The tissue levels of IL-1β and TNF-α (pg/mg protein) were determined using a standard curve.

### Behavioral tests

#### Passive avoidance test

This device had a light and dark compartment separated by a guillotine door. The electric shock was applied to the steel conductive grid at the bottom of the device. This test was performed for three consecutive days. On the first day, the animals were allowed to explore the apparatus for 5 min. On the second day, the acquisition test was performed. The rats were left in the light compartment and after 2 min of adaptation, the guillotine door was opened and allowed the rat entered to the dark compartment, after that, the door was closed and the animal was given an electric shock (1.5 mA for 3 s). 24 h later, each rat was placed in the light chamber and again after 2 min of adaptation, the guillotine door was opened and allowed the rats to enter to the dark compartment without any shock induction. In this phase, the latency time (step-through latency) and the time spent in the dark chamber was recorded (up to 300 s)^17^.

#### Novel object test

The novel object recognition test is a simple memory assessment that does not require any external motivation, reward, or punishment and relies only on the explorative behavior of rodents.

In this study, the test included three stages and it was done in a quiet environment, without any noise and with a constant light. In the first stage (habituation), which is to acquaint the animal with the environment, the animal was placed in the test room and allowed to explore the area freely for 5 min. At this stage, no object was placed in the room. After searching for a while, the animal was returned to its cage. In the second stage (training stage), two completely identical objects in terms of appearance, shape, color, and texture were placed in opposite corners of the room at a distance of 10 cm from the wall. The rat was given some time to explore the objects. After some time, the rat was returned to its cage. In the third stage (testing stage), which is the animal's memory test, one of the objects in the room was replaced with a new object that had different appearance characteristics.

In the Novel Object Recognition Test, the duration of the rat's exploration of the objects during the training and testing stages was 5 min, during which the rat's interactions with the objects are observed and recorded.

The interval between the habituation and training stages in the novel object recognition test was 24 h. This allows the rats to become familiar with the testing environment and reduces any potential stress or novelty-induced anxiety before the training phase. Similarly, the interval between the training and testing stages was 24 h as well. This delay allows for memory consolidation to occur and ensures that the rats are tested on their ability to recognize the novel object rather than simply remembering it from a recent encounter.

In the NORT, two criteria were recorded: Difference score and Recognition index. For the difference score, the average difference time of examining the novel object and the familiar object, was recorded. Normal rats spent more time around the new object compared to the familiar object. To evaluate the recognition index; the percentage of time spent around the new object to the total time around both objects, was recorded^18^.

#### Rotarod

The rotarod device was used to check the balance activities and the power of motor coordination between the limbs. To evaluate the balance activity by this device, the animal was placed on a rotating horizontal bar with a diameter of 5 cm, speed was increased from 5 to 45 rpm in 300 s, and the time to maintain balance was recorded. First, each animal was given two opportunities to adapt to the device, and then the animal was placed on the rotating rod three times with 2-min intervals, and the average of these times was calculated. It should be noted that the duration of the rat's stay on the rod was determined to be a maximum of 300 s^19^.

#### Wire hanging test

Wire hanging test assesses multiple aspects of locomotor ability, including grip strength, endurance, and body cooperation. It is widely used in rodents with neurological disorders and/or muscle weakness. Animals are placed on a wire hanging 50–60 cm above the ground for a maximum of 2 min, so that they have to suspend their bodies with limbs. The time animals spend on the wire (latency before falling), which reflects muscle strength, is recorded. While hanging, animals might use forelimbs or all four limbs to hold the wire^20^.

#### Open-field test (OFT)

In this test, the motor activity of the animal, based on moving from one point to another, is measured and calculated by a camera. This device consists of an open cube box with a wooden floor and plexiglass walls measuring 50 × 50 × 50 cm. After setting the device and cleaning the inner wall of the cubic box, the animal is placed in the center of the box and the first 5 min are considered for the animals to be familiar with OFT. Then the total traveled distance, mean velocity, rearing frequency (the number of standing on two hind limbs), and grooming number were recorded for 5 minute^21^.

### Statistical analysis

All data are presented as mean ± SEM. statistical analysis and graphs were done using Prism software and considering the significance level of *p* < 0.05. The statistical difference of different parameters among different groups was done using a one-way analysis of variance and Tukey's post-test. To compare inflammatory factors between groups in two time periods, a two-way repeated measure analysis of variance, was used.

## Results

### Passive avoidance task results

The step through latency in the passive avoidance test in the hypoxia group was lower than the control group (*p* < 0.05). But alpha-pinene 5 and 10 mg/kg without hypoxia increased the step through latency compared to the control group (*p* < 0.001). The latency time entering to the dark compartment in the hypoxia group treated with alpha-pinene 5 and 10 mg/kg significantly increased compared to the hypoxia group (*p* < 0.05) (Fig. 2A).

The time spent in the dark compartment in the hypoxia group was significantly higher than the control group (*p* < 0.001). In the alpha-pinene 5 and 10 mg/kg without hypoxia, the time spent in the dark compartment significantly decreased compared to the control group (*p* < 0.05). Time spent in the dark compartment significantly decreased in the hypoxia groups treated with alpha-pinene 5 and 10 mg/kg compared to the hypoxia group (*p* < 0.05) (Fig. 2B).

### Novel object recognition test results

**Figure 3:**
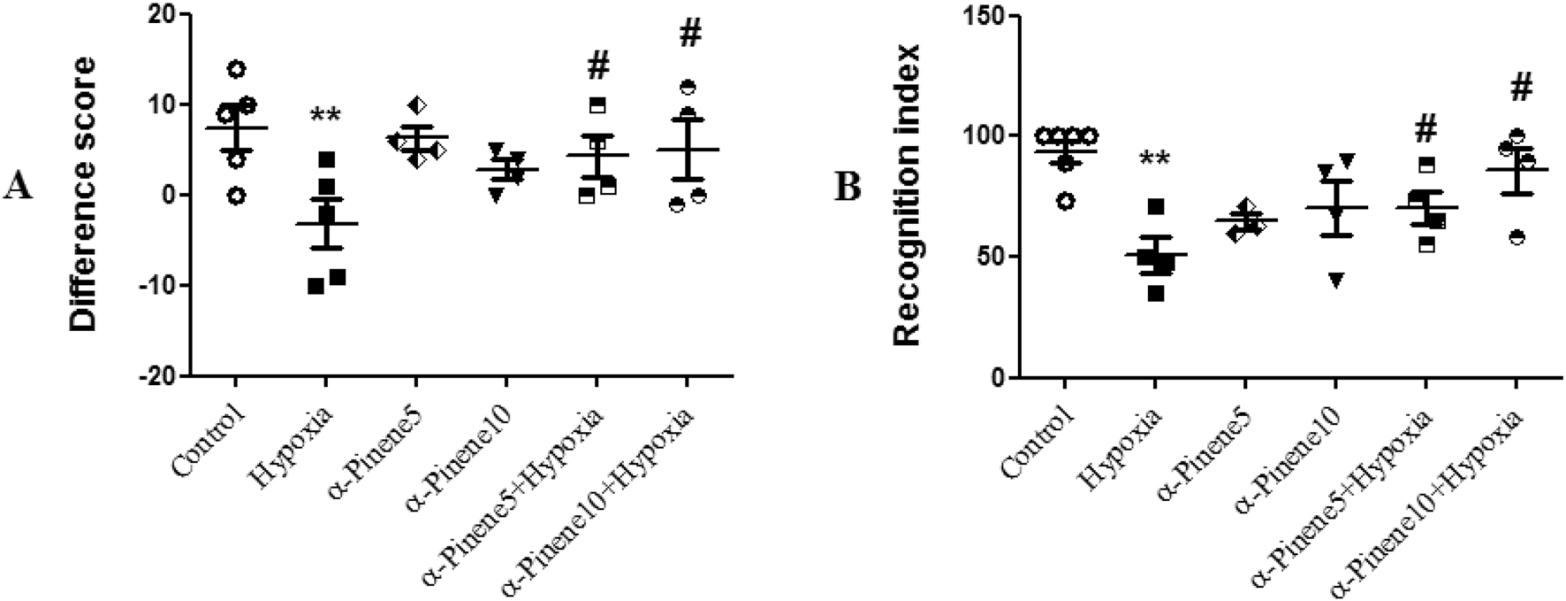
Comparison the average of (**A**) Difference score and (**B**) Recognition index between the studied groups. ** *p* < 0.01 indicates a significant difference between experimental groups versus Control group. # *p* < 0.05 shows a significant difference between the hypoxia groups treated with alpha-pinenes and the hypoxia group (n = 6). Data represent as the mean ± SEM.

As shown in Fig. 3, the average time difference between new and old objects in most of the hypoxia rats was under zero and close to negative values, except in two cases. In most of the rats in the experimental groups, the data were evaluated as more than zero and positive. According to the results, the rats in the hypoxia group spent less time around the new object and more time near the old one compared to the control group (*p* < 0.01), while the time spent around the new object was higher than the old object in the hypoxia groups treated with alpha-pinene 5 and 10 mg/kg compared to the hypoxia group (*p* < 0.05) (Fig. 3A).

The percentage of time spent around the new object to the total time around both objects was under 50% in most of the rats in the hypoxia group and more than 50% in most of the rats in the experimental groups. The recognition index in the hypoxia group significantly decreased compared to the control group (*p* < 0.01). This index significantly increased in both hypoxic rats treated with alpha-pinene compared to the hypoxia group (*p* < 0.05) (Fig. 3B).

### Rotarod results

**Figure 4:**
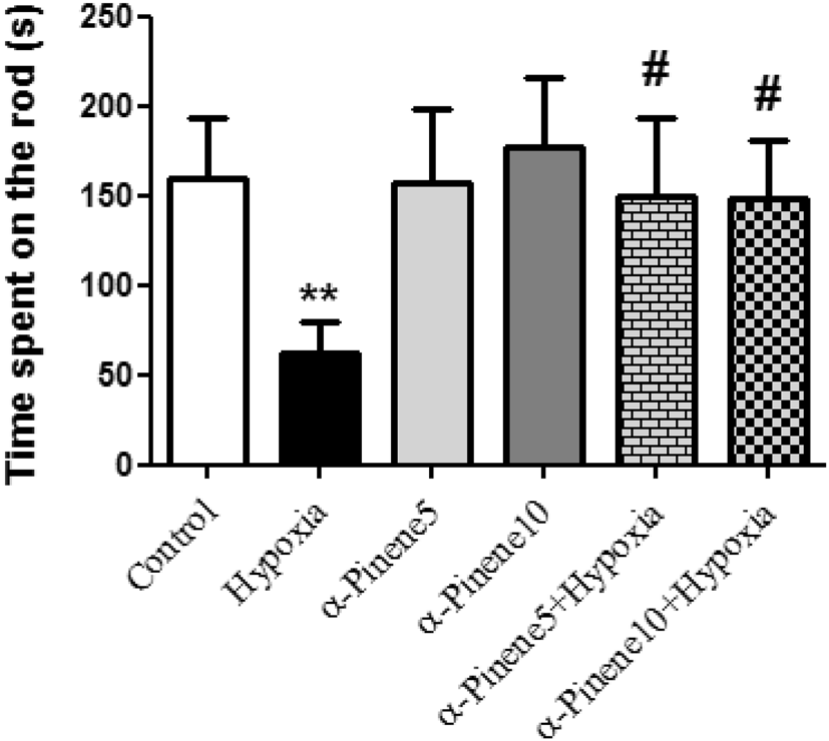
The mean of time staying on the rotarod between the studied groups. ** *p* < 0.01 indicates a significant difference between experimental groups versus Control group. # *p* < 0.05 shows a significant difference between the hypoxia groups treated with alpha-pinenes and the hypoxia group (n = 6). Data represent as the mean ± SEM.

According to the results, there was a significant difference in the average time spent on the rod between the hypoxia and control group, and rats in the hypoxia group spent less time on the rotating rod compared to the control group (*p* < 0.01). The average time spent on the rotating rod and balance maintenance in the treatment groups was significantly more than the hypoxia group (*p* < 0.05) (Fig. 4).

### The result of the wire hanging test

**Figure 5:**
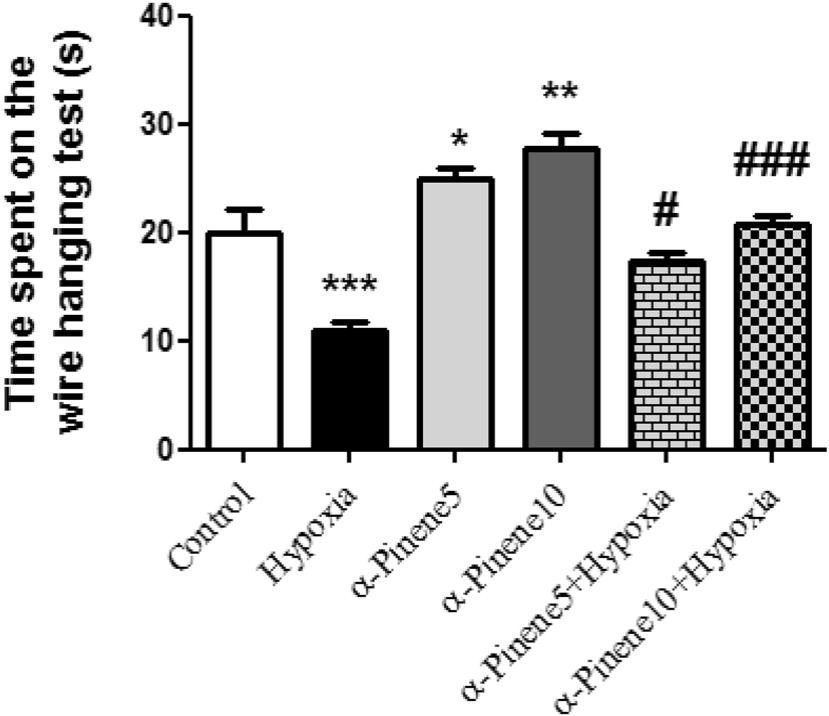
Comparison the average of time spent on the wire hanging test. *** *p* < 0.001 and * *p* < 0.05 indicates a significant difference between experimental groups versus Control group. # *p* < 0.05 and ### *p* < 0.001 shows a significant difference between the hypoxia groups treated with alpha-pinenes and the hypoxia group (n = 6). Data represent as the mean ± SEM.

As shown in Fig. 5, the alpha-pinene (10mg/kg) group had the most average time to maintain balance on the inverted grid in the wire hanging test and the smallest time was related to the hypoxia group. Maintenance time in the hypoxia group significantly decreased compared to the control group (*p* < 0.001). But this time in the groups received alpha-pinene 5 (*p* < 0.05) and 10 mg/kg (*p* < 0.01) significantly increased compared to the control group. In addition, a significant increase was seen in the hypoxic rats treated with alpha-pinene 5 (*p* < 0.05) and 10 mg/kg (*p* < 0.001) compared to the hypoxia group (Fig. 5).

### The open field results

This test is used to investigate motor activity and exploratory behavior in rodents. In this test, the average number of times of standing on the hind legs (rearing), self-treatment (grooming), the average traveled distance in the open field box and the movement velocity, were measured.

**Figure 6:**
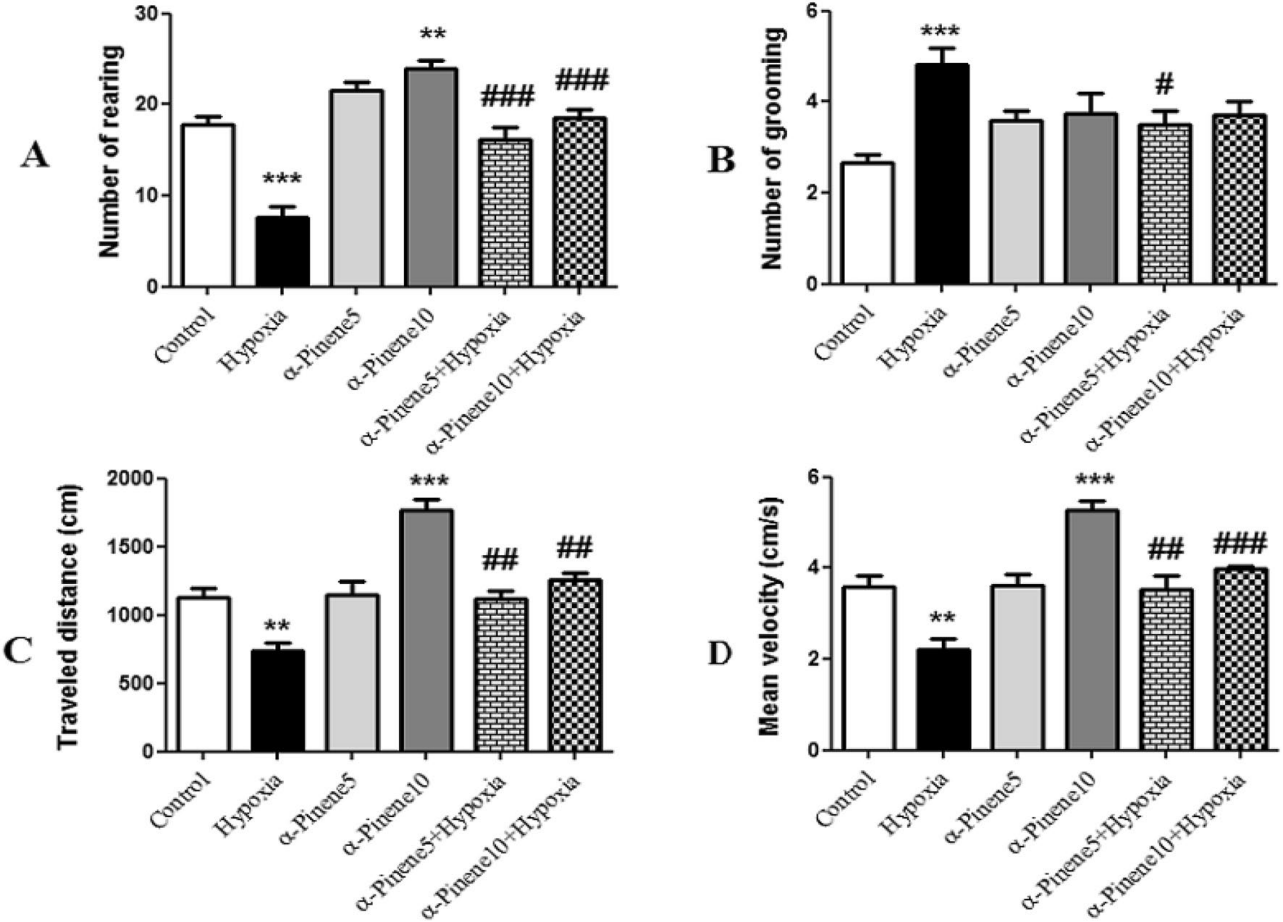
The average of (**A**) Rearing, (**B**) Grooming, (**C**) Traveled distance (cm), (**D**) Velocity (cm/s) in the open field test. ** *p* < 0.01, *** *p* < 0.001 indicates a significant difference between experimental groups versus Control group. # *p* < 0.05, ## *p* < 0.01, ### *p* < 0.001 shows a significant difference between the hypoxia groups treated with alpha-pinenes and the hypoxia group (n = 6). Data represent as the mean ± SEM.

According to the result, the number of rearing in the rats treated with alpha-pinene-10 was significantly higher than in the control group (*p* < 0.01) while rearing frequency in the hypoxia group was significantly reduced compared to the control group (*p* < 0.001). The difference between hypoxic rats treated with alpha-pinene 5 and 10 mg/kg and the hypoxia group was significant and the number of rearing in the hypoxia groups treated with alpha-pinene increased compared to the hypoxia group (*p* < 0.001) (Fig. 6A). In rodents, grooming activity and its microstructure may serve as a useful measure of stress and anxiety in both wild and laboratory animals. As shown in Fig. 6B, the number of grooming in the hypoxia group was higher than in the control group (*p* < 0.001). Also, the number of times of self-treatment in the hypoxia group treated with alpha-pinene 5 showed a significant decrease compared to the hypoxia group (*p* < 0.05). No significant difference was observed among other groups (Fig. 6B).

The results of the total traveled distance in this test showed that the hypoxia group traveled less distance than the control group in the open field box (*p* < 0.01). The experimental group of alpha-pinene 10 also covered more distance and there was a significant difference compared to the control group (*p* < 0.001). In further investigations, a significant difference was seen between the hypoxia groups treated with alpha-pinene 5 and 10 mg/kg compared to hypoxia group, and the average of traveled distance in these groups was significantly increased compared to the hypoxia group (*p* < 0.01) (Fig. 6C).

Movement velocity in the alpha-pinene 10 mg/kg significantly increased compared to the control group (*p* < 0.001). But, the mean velocity in the hypoxia group was lower than in the control group (*p* < 0.01). The average speed of movement in the hypoxia group treated with alpha-pinene 5 (*p* < 0.01) and 10 (*p* < 0.001) also increased significantly compared to the hypoxia group (Fig. 6D).

### TNF-α assay

**Figure 7:**
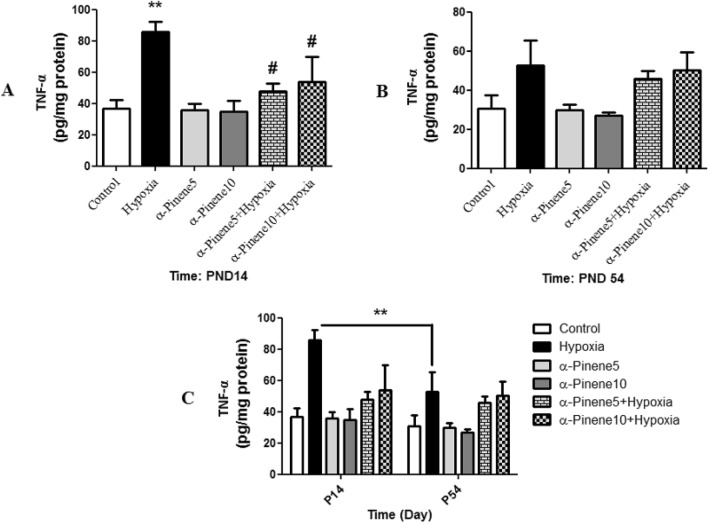
Comparison the level of TNF-α between groups at (**A**) PND14, (**B**) PND14, ** *p* < 0.01, indicates a significant difference between experimental groups versus Control group. # *p* < 0.05 shows a significant difference between the hypoxia groups treated with alpha-pinenes and the hypoxia group (n = 6). (**C**) Shows the difference between each group at PND14 and PND54. ** *p* < 0.01indicates a significant difference between hypoxia groups at PND54 compared to the PND14 (n = 6). Data represent as the mean ± SEM.

Pro-inflammatory cytokines were measured in rats of different ages: at the postnatal day 14 (PND14; 24 h after the last hypoxia session) and at PND54 (after behavior tests). Then these results were analyzed and compared with each other by two-way ANOVA. Our results demonstrated that in a rat model of hypoxia the production of pro-inflammatory cytokines, TNF-α was higher than the control group at PND14 (*p* < 0.01) and in the hypoxia group treated with alpha-pinene 5 and 10 mg/kg significantly decreased compared to the hypoxia group (*p* < 0.05) (Fig. 7A). The difference between groups at PND54 was not significant (Fig. 7B). Two-way analysis showed that TNF-**α** in the hypoxia group at PND 54 was lower than the hypoxia group at PND14 (*p* < 0.01) (Fig. 7C).

### The result of IL-1β level

**Figure 8:**
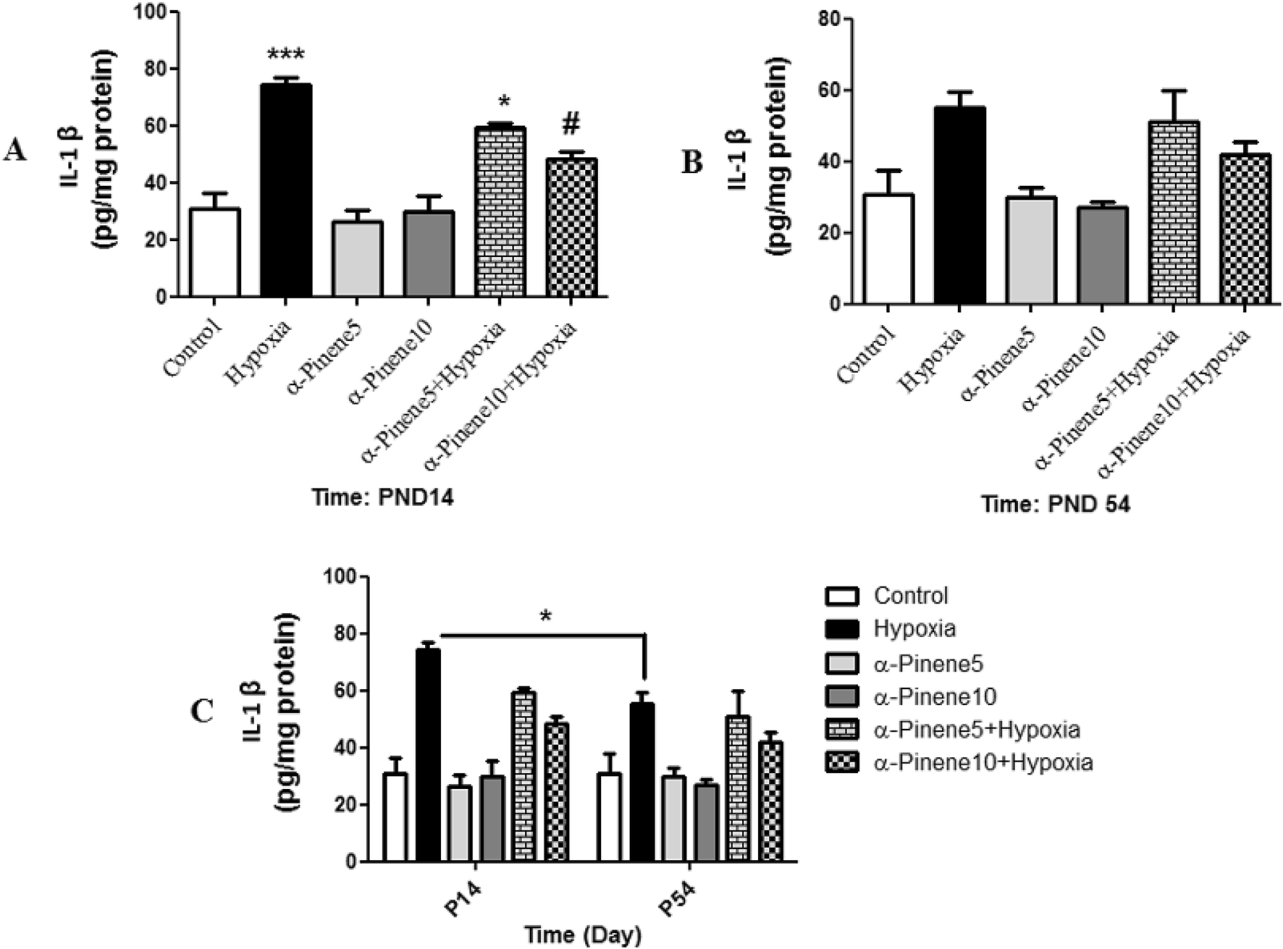
Comparison the level of IL-1β between groups at (**A**) PND14, (**B**) PND14, * *p* < 0.05 and *** *p* < 0.001, indicates a significant difference between experimental groups versus Control group. # *p* < 0.05 shows a significant difference between the hypoxia groups treated with alpha-pinenes and the hypoxia group (n = 6). (**C**) Shows the difference between each group at PND14 and PND54. * *p* < 0.05 indicates a significant difference between hypoxia groups at PND54 compared to the PND14 (n = 6). Data represent as the mean ± SEM.

The levels of IL-1β in the hypoxia group (*p* < 0.001) and hypoxia group treated with alpha-pinene 5 mg/kg (*p* < 0.05) were higher than the control group. However IL-1β level in the hypoxia group treated with alpha-pinene 10 mg/kg was significantly decreased compared to the hypoxia group at PND14 (*p* < 0.05) (Fig. 8A). The difference between groups at PND54 was not significant (Fig. 8B). Two-way analysis showed that IL-1β in the hypoxia group at PND 54 was lower than the hypoxia group at PND14 (*p* < 0.05) (Fig. 8C).

## Discussion

Prenatal hypoxia, which can occur due to abnormal pregnancy or maternal infection, has been shown to impact the developmental and behavioral characteristics of animals. Studies have demonstrated memory impairment, motor dysfunction, and anxiety symptoms in animal models of prenatal hypoxia^22–25^. Hypoxia ischemia (HI), characterized by reduced oxygen and/or blood flow to the brain, is a common injury among preterm and term infants with birth complications, leading to cognitive and behavioral deficits^26^. In the current study, behavioral performance including; passive avoidance memory, cognition, balance maintenance and motor activity was impaired in the hypoxia group compared to the control group.

In recent years, to better understand the effect of hypoxia on brain development and the molecular mechanism in offspring; animal models of prenatal hypoxia have been widely used and reviewed, including rats, mice, rabbits, chickens, and sheep. Rats and mice are the most popular and convenient rodent models, and both can be used in prenatal and early postnatal hypoxia studies^27^.

Several experimental and clinical studies have shown the effect of hypoxia on behavioral functions. Liu et al., reported that neonatal hypoxia–ischemia (HI) produces neurodegeneration and brain injury, and leads to behavioral and cognitive dysfunction^28^. Hill et al., data also indicated significant deficits in rapid auditory processing and spatial learning in male rats with postnatal day 1 (P1), P7, or P10 hypoxia injury^29^. In another study motor ability was assessed in 25 children born at term with perinatal hypoxia and results showed that, eight of 25 children had cerebral palsy (CP), and in another 17 children without CP, nine of them had impaired motor ability and four had behavioral problems^30^. Studies have also shown that neonatal hypoxia can lead to neurodegeneration, brain injury, and behavioral dysfunction.

Hypoxia can induce widespread behavioral problems by increasing oxidative stress and inflammation, affecting synaptic maturation and leading to brain damage.

Neuroinflammation following hypoxia leads to brain damage and activation of glial cells, which produce inflammatory cytokines such as interleukin 1-β (IL-1β) and tumor necrosis factor-alpha (TNF-α).

A large number of cytokine receptors are expressed in the hippocampus, an important area in cognitive function, so it can be affected by inflammation induced by brain hypoxia^31^.

One possible mechanism for behavioral impairment following hypoxia is increased oxidative stress and inflammation. Therefore, the application of anti-inflammatory compounds may be useful in mitigating the effects of hypoxia. Alpha-pinene treatment has shown potential as a neuroprotective agent in hypoxia injury, with studies demonstrating improvement in behavioral responses in animals treated with alpha-pinene compared to those exposed to hypoxia alone.

Some studies have investigated the effect of alpha-pinene on normoxic rats, which are rats that are exposed to normal levels of oxygen. For example; alpha-pinene modulates the inflammatory response and protects against brain ischemia–reperfusion injury in normoxic rats by inhibiting the inducible nitric oxide synthase (iNOS)-nuclear factor kappa B (NF-κB)-cyclooxygenase-2 (COX-2) pathway and reducing caspase-3 activation^32^. Alpha-pinene enhances the gastric mucosal defense and reduces the ethanol-induced gastric damage in normoxic rats by upregulating the nuclear factor erythroid 2-related factor 2 (Nrf2) and heme oxygenase-1 (HO-1) expression^33^ and alpha-pinene does not cause significant toxicity or carcinogenicity in normoxic rats when administered by inhalation for up to 2 years^34^. These studies suggest that alpha-pinene has beneficial effects on the brain and stomach of normoxic rats, and that it is relatively safe to use. However, more research is needed to confirm the mechanisms and the optimal doses of alpha-pinene for different conditions.

Previous studies have also demonstrated that alpha-pinene can have a positive impact on learning and memory deficits in animal models of neurodegenerative diseases^35^.

Additionally, alpha-pinene has been found to improve memory deficits in amnesia-related models and enhance learning and memory in normal rats^36^. It has also been shown to increase spatial memory and improve memory performance in the acquisition phase of the Morris water maze test^37^. In a rat model of Parkinson's disease, alpha-pinene was shown to have protective effects against memory impairment by regulating antioxidative and anti-acetylcholinesterase mechanisms and enhancing dopamine concentration^38^. Additionally, alpha-pinene administration for 2 weeks has been found to significantly enhance memory performance by decreasing MDA levels and increasing thiol concentration in the hippocampus^39^. A study conducted by Kasuya and colleagues explored the impact of Chamaecyparis obtusa essential oil, which is a rich source of alpha-pinene, on emotional behavior and motor activity. The researchers discovered that alpha-pinene is present in various regions of the brain and has a dose-dependent anxiolytic or excitatory effect. They also suggested that in addition to the striatum and hippocampus, other brain areas are involved in the increased motor activity caused by high levels of alpha-pinene^40^.

The inflammation and immunological pathway involves the production of cytokines, such as interleukin-1β, tumor necrosis factor-α (TNF-α), and IL-6, by various cells in the central nervous system (CNS), including microglia, astrocytes, and neurons. These cytokines play a significant role in both normal CNS development and the response to brain injury. Studies have shown that elevated levels of IL-6, IL-10, and TNF-α in cerebrospinal fluid (CSF) may be associated with brain injury in both neonatal hypoxic-ischemic encephalopathy (HIE) and preterm infants with MRI-defined brain white matter injury. Furthermore, hypoxic ischemia (HI) in pregnant rats resulted in elevated levels of IL-1β and IL-6 mRNA and protein in the offspring's brain and blood samples. The activation of the NF-κB pathway in neurons and glial cells has also been observed in response to asphyxial injury, leading to the induction of genes associated with the innate immune response^41–44^.

Alpha-pinene has a strong inhibitory effect on inflammation in pathological conditions. It can suppress microglial cells and inhibit the production of inflammatory cytokines such as IL-1β, NF-κB, and LTB4^45,46^. In our study, in the neonatal hypoxia group, the production of pro-inflammatory cytokines TNF-α and IL-1β was higher than the control group at PND14, but the levels were decreased in the hypoxia group treated with alpha-pinene at 5 and 10 mg/kg. Also, the two-way analysis showed that TNF-α and IL-1β in the hypoxia group at PND 54 were lower than the hypoxia group at PND14. This suggests that alpha-pinene may have potential as a therapeutic agent for reducing inflammation associated with hypoxic conditions (Supplementary Figure).

## Conclusion

Based on the results of the study, it is evident that neonatal hypoxia leads to impaired behavioral functions and increased inflammation in rats. However, the administration of alpha-pinene during hypoxia showed significant improvement in behavioral functions and a decrease in inflammatory factors compared to the hypoxia group. Therefore, according to these findings, it can be concluded that alpha-pinene may serve as a protective factor in reducing inflammation in hypoxia-induced brain injury. This suggests that alpha-pinene has the potential to mitigate the detrimental effects of hypoxia on behavioral and cognitive functions.

### Supplementary Information

Supplementary Information.

Supplementary Information.

## Data Availability

The datasets used and/or analyzed during the current study are available from the corresponding author on reasonable request.
