# Supplementary material for: Alpha-pinene ameliorate behavioral deficit induced by early postnatal hypoxia in the rat: study the inflammatory mechanism
Source: Sci Rep. 2024 Mar 17;14:6416. doi: 10.1038/s41598-024-56756-1 (PMC10944845; doi:10.1038/s41598-024-56756-1)
Supplement: Supplementary file 1 — Supplementary Information. [file 41598_2024_56756_MOESM1_ESM.docx]

| **Tests of Within-Subjects Effects** | | | | | | |
| --- | --- | --- | --- | --- | --- | --- |
| Measure:MEASURE_1 | |  |  |  |  |  |
| Source | | Type III Sum of Squares | df | Mean Square | F | Sig. |
| factor1 | Sphericity Assumed | 793.317 | 1 | 793.317 | 7.214 | .013 |
|  | Greenhouse-Geisser | 793.317 | 1.000 | 793.317 | 7.214 | .013 |
|  | Huynh-Feldt | 793.317 | 1.000 | 793.317 | 7.214 | .013 |
|  | Lower-bound | 793.317 | 1.000 | 793.317 | 7.214 | .013 |
| factor1 * groups | Sphericity Assumed | 899.530 | 5 | 179.906 | 1.636 | .189 |
|  | Greenhouse-Geisser | 899.530 | 5.000 | 179.906 | 1.636 | .189 |
|  | Huynh-Feldt | 899.530 | 5.000 | 179.906 | 1.636 | .189 |
|  | Lower-bound | 899.530 | 5.000 | 179.906 | 1.636 | .189 |
| Error(factor1) | Sphericity Assumed | 2639.239 | 24 | 109.968 |  |  |
|  | Greenhouse-Geisser | 2639.239 | 24.000 | 109.968 |  |  |
|  | Huynh-Feldt | 2639.239 | 24.000 | 109.968 |  |  |
|  | Lower-bound | 2639.239 | 24.000 | 109.968 |  |  |

| **Tests of Between-Subjects Effects** | | | | | |
| --- | --- | --- | --- | --- | --- |
| Measure:MEASURE_1  Transformed Variable:Average | | |  |  |  |
| Source | Type III Sum of Squares | df | Mean Square | F | Sig. |
| Intercept | 109428.436 | 1 | 109428.436 | 755.002 | .000 |
| groups | 11637.446 | 5 | 2327.489 | 16.059 | .000 |
| Error | 3478.513 | 24 | 144.938 |  |  |

| **Estimates** | | | | |
| --- | --- | --- | --- | --- |
| Measure:MEASURE_1 |  |  |  |  |
| groups | Mean | Std. Error | 95% Confidence Interval | |
|  |  |  | Lower Bound | Upper Bound |
| Control | 29.953 | 3.807 | 22.096 | 37.811 |
| Hypoxia | 65.671 | 3.807 | 57.814 | 73.528 |
| Alpha-pinene5 | 28.245 | 3.807 | 20.387 | 36.102 |
| Alpha-pinene10 | 32.041 | 3.807 | 24.184 | 39.899 |
| Alpha-pinene5+Hypoxia | 54.615 | 3.807 | 46.758 | 62.473 |
| Alpha-pinene10+Hypoxia | 45.711 | 3.807 | 37.853 | 53.568 |

| **Pairwise Comparisons** | | | | | | |
| --- | --- | --- | --- | --- | --- | --- |
| Measure:MEASURE_1 |  |  |  |  |  |  |
| (I) groups | (J) groups | Mean Difference (I-J) | Std. Error | Sig.^a^ | 95% Confidence Interval for Difference^a^ | |
|  |  |  |  |  | Lower Bound | Upper Bound |
| Control | Hypoxia | -35.718^*^ | 5.384 | .000 | -46.830 | -24.606 |
|  | Alpha-pinene5 | 1.709 | 5.384 | .754 | -9.403 | 12.821 |
|  | Alpha-pinene10 | -2.088 | 5.384 | .702 | -13.200 | 9.024 |
|  | Alpha-pinene5+Hypoxia | -24.662^*^ | 5.384 | .000 | -35.774 | -13.550 |
|  | Alpha-pinene10+Hypoxia | -15.757^*^ | 5.384 | .007 | -26.869 | -4.645 |
| Hypoxia | Control | 35.718^*^ | 5.384 | .000 | 24.606 | 46.830 |
|  | Alpha-pinene5 | 37.426^*^ | 5.384 | .000 | 26.314 | 48.539 |
|  | Alpha-pinene10 | 33.630^*^ | 5.384 | .000 | 22.518 | 44.742 |
|  | Alpha-pinene5+Hypoxia | 11.056 | 5.384 | .051 | -.056 | 22.168 |
|  | Alpha-pinene10+Hypoxia | 19.960^*^ | 5.384 | .001 | 8.848 | 31.072 |
| Alpha-pinene5 | Control | -1.709 | 5.384 | .754 | -12.821 | 9.403 |
|  | Hypoxia | -37.426^*^ | 5.384 | .000 | -48.539 | -26.314 |
|  | Alpha-pinene10 | -3.797 | 5.384 | .487 | -14.909 | 7.315 |
|  | Alpha-pinene5+Hypoxia | -26.371^*^ | 5.384 | .000 | -37.483 | -15.259 |
|  | Alpha-pinene10+Hypoxia | -17.466^*^ | 5.384 | .003 | -28.578 | -6.354 |
| Alpha-pinene10 | Control | 2.088 | 5.384 | .702 | -9.024 | 13.200 |
|  | Hypoxia | -33.630^*^ | 5.384 | .000 | -44.742 | -22.518 |
|  | Alpha-pinene5 | 3.797 | 5.384 | .487 | -7.315 | 14.909 |
|  | Alpha-pinene5+Hypoxia | -22.574^*^ | 5.384 | .000 | -33.686 | -11.462 |
|  | Alpha-pinene10+Hypoxia | -13.669^*^ | 5.384 | .018 | -24.781 | -2.557 |
| Alpha-pinene5+Hypoxia | Control | 24.662^*^ | 5.384 | .000 | 13.550 | 35.774 |
|  | Hypoxia | -11.056 | 5.384 | .051 | -22.168 | .056 |
|  | Alpha-pinene5 | 26.371^*^ | 5.384 | .000 | 15.259 | 37.483 |
|  | Alpha-pinene10 | 22.574^*^ | 5.384 | .000 | 11.462 | 33.686 |
|  | Alpha-pinene10+Hypoxia | 8.904 | 5.384 | .111 | -2.208 | 20.016 |
| Alpha-pinene10+Hypoxia | Control | 15.757^*^ | 5.384 | .007 | 4.645 | 26.869 |
|  | Hypoxia | -19.960^*^ | 5.384 | .001 | -31.072 | -8.848 |
|  | Alpha-pinene5 | 17.466^*^ | 5.384 | .003 | 6.354 | 28.578 |
|  | Alpha-pinene10 | 13.669^*^ | 5.384 | .018 | 2.557 | 24.781 |
|  | Alpha-pinene5+Hypoxia | -8.904 | 5.384 | .111 | -20.016 | 2.208 |
| Based on estimated marginal means | |  |  |  |  |  |
| *. The mean difference is significant at the .05 level. | | |  |  |  |  |
| a. Adjustment for multiple comparisons: Least Significant Difference (equivalent to no adjustments). | | | | | |  |

| **Paired Samples Test** | | | | | | | | | | |
| --- | --- | --- | --- | --- | --- | --- | --- | --- | --- | --- |
| groups | | | Paired Differences | | | | | t | df | Sig. (2-tailed) |
|  |  |  | Mean | Std. Deviation | Std. Error Mean | 95% Confidence Interval of the Difference | |  |  |  |
|  |  |  |  |  |  | Lower | Upper |  |  |  |
| Control | Pair 1 | PND14 - PND54 | 1.02920 | 17.24977 | 7.71433 | -20.38922 | 22.44762 | .133 | 4 | .900 |
| Hypoxia | Pair 1 | PND14 - PND54 | 2.30119E1 | 13.74611 | 6.14745 | 5.94380 | 40.07991 | 3.743 | 4 | .020 |
| Alpha-pinene5 | Pair 1 | PND14 - PND54 | .36809 | 6.11218 | 2.73345 | -7.22117 | 7.95736 | .135 | 4 | .899 |
| Alpha-pinene10 | Pair 1 | PND14 - PND54 | 2.48121 | 10.16276 | 4.54492 | -10.13752 | 15.09994 | .546 | 4 | .614 |
| Alpha-pinene5+Hypoxia | Pair 1 | PND14 - PND54 | 8.19767 | 20.28878 | 9.07342 | -16.99417 | 33.38951 | .903 | 4 | .417 |
| Alpha-pinene10+Hypoxia | Pair 1 | PND14 - PND54 | 8.54635 | 16.75811 | 7.49446 | -12.26159 | 29.35430 | 1.140 | 4 | .318 |

| **Tests of Within-Subjects Effects** | | | | | | |
| --- | --- | --- | --- | --- | --- | --- |
| Measure:MEASURE_1 | |  |  |  |  |  |
| Source | | Type III Sum of Squares | df | Mean Square | F | Sig. |
| factor1 | Sphericity Assumed | 1480.682 | 1 | 1480.682 | 13.835 | .001 |
|  | Greenhouse-Geisser | 1480.682 | 1.000 | 1480.682 | 13.835 | .001 |
|  | Huynh-Feldt | 1480.682 | 1.000 | 1480.682 | 13.835 | .001 |
|  | Lower-bound | 1480.682 | 1.000 | 1480.682 | 13.835 | .001 |
| factor1 * groups | Sphericity Assumed | 1144.644 | 5 | 228.929 | 2.139 | .095 |
|  | Greenhouse-Geisser | 1144.644 | 5.000 | 228.929 | 2.139 | .095 |
|  | Huynh-Feldt | 1144.644 | 5.000 | 228.929 | 2.139 | .095 |
|  | Lower-bound | 1144.644 | 5.000 | 228.929 | 2.139 | .095 |
| Error(factor1) | Sphericity Assumed | 2568.501 | 24 | 107.021 |  |  |
|  | Greenhouse-Geisser | 2568.501 | 24.000 | 107.021 |  |  |
|  | Huynh-Feldt | 2568.501 | 24.000 | 107.021 |  |  |
|  | Lower-bound | 2568.501 | 24.000 | 107.021 |  |  |

| **Tests of Between-Subjects Effects** | | | | | |
| --- | --- | --- | --- | --- | --- |
| Measure:MEASURE_1  Transformed Variable:Average | | |  |  |  |
| Source | Type III Sum of Squares | df | Mean Square | F | Sig. |
| Intercept | 123148.358 | 1 | 123148.358 | 711.424 | .000 |
| groups | 11516.198 | 5 | 2303.240 | 13.306 | .000 |
| Error | 4154.427 | 24 | 173.101 |  |  |

| **Estimates** | | | | |
| --- | --- | --- | --- | --- |
| Measure:MEASURE_1 |  |  |  |  |
| groups | Mean | Std. Error | 95% Confidence Interval | |
|  |  |  | Lower Bound | Upper Bound |
| Control | 36.075 | 4.161 | 27.488 | 44.662 |
| Hypoxia | 70.671 | 4.161 | 62.084 | 79.258 |
| Alpha-pinene5 | 32.945 | 4.161 | 24.358 | 41.531 |
| Alpha-pinene10 | 31.041 | 4.161 | 22.454 | 39.628 |
| Alpha-pinene5+Hypoxia | 48.115 | 4.161 | 39.528 | 56.702 |
| Alpha-pinene10+Hypoxia | 52.978 | 4.161 | 44.391 | 61.565 |

| **Pairwise Comparisons** | | | | | | |
| --- | --- | --- | --- | --- | --- | --- |
| Measure:MEASURE_1 |  |  |  |  |  |  |
| (I) groups | (J) groups | Mean Difference (I-J) | Std. Error | Sig.^a^ | 95% Confidence Interval for Difference^a^ | |
|  |  |  |  |  | Lower Bound | Upper Bound |
| Control | Hypoxia | -34.596^*^ | 5.884 | .000 | -46.739 | -22.452 |
|  | Alpha-pinene5 | 3.131 | 5.884 | .600 | -9.013 | 15.275 |
|  | Alpha-pinene10 | 5.034 | 5.884 | .401 | -7.110 | 17.178 |
|  | Alpha-pinene5+Hypoxia | -12.040 | 5.884 | .052 | -24.184 | .104 |
|  | Alpha-pinene10+Hypoxia | -16.902^*^ | 5.884 | .008 | -29.046 | -4.759 |
| Hypoxia | Control | 34.596^*^ | 5.884 | .000 | 22.452 | 46.739 |
|  | Alpha-pinene5 | 37.726^*^ | 5.884 | .000 | 25.583 | 49.870 |
|  | Alpha-pinene10 | 39.630^*^ | 5.884 | .000 | 27.486 | 51.773 |
|  | Alpha-pinene5+Hypoxia | 22.556^*^ | 5.884 | .001 | 10.412 | 34.700 |
|  | Alpha-pinene10+Hypoxia | 17.693^*^ | 5.884 | .006 | 5.549 | 29.837 |
| Alpha-pinene5 | Control | -3.131 | 5.884 | .600 | -15.275 | 9.013 |
|  | Hypoxia | -37.726^*^ | 5.884 | .000 | -49.870 | -25.583 |
|  | Alpha-pinene10 | 1.903 | 5.884 | .749 | -10.241 | 14.047 |
|  | Alpha-pinene5+Hypoxia | -15.171^*^ | 5.884 | .016 | -27.314 | -3.027 |
|  | Alpha-pinene10+Hypoxia | -20.033^*^ | 5.884 | .002 | -32.177 | -7.890 |
| Alpha-pinene10 | Control | -5.034 | 5.884 | .401 | -17.178 | 7.110 |
|  | Hypoxia | -39.630^*^ | 5.884 | .000 | -51.773 | -27.486 |
|  | Alpha-pinene5 | -1.903 | 5.884 | .749 | -14.047 | 10.241 |
|  | Alpha-pinene5+Hypoxia | -17.074^*^ | 5.884 | .008 | -29.218 | -4.930 |
|  | Alpha-pinene10+Hypoxia | -21.936^*^ | 5.884 | .001 | -34.080 | -9.793 |
| Alpha-pinene5+Hypoxia | Control | 12.040 | 5.884 | .052 | -.104 | 24.184 |
|  | Hypoxia | -22.556^*^ | 5.884 | .001 | -34.700 | -10.412 |
|  | Alpha-pinene5 | 15.171^*^ | 5.884 | .016 | 3.027 | 27.314 |
|  | Alpha-pinene10 | 17.074^*^ | 5.884 | .008 | 4.930 | 29.218 |
|  | Alpha-pinene10+Hypoxia | -4.863 | 5.884 | .417 | -17.006 | 7.281 |
| Alpha-pinene10+Hypoxia | Control | 16.902^*^ | 5.884 | .008 | 4.759 | 29.046 |
|  | Hypoxia | -17.693^*^ | 5.884 | .006 | -29.837 | -5.549 |
|  | Alpha-pinene5 | 20.033^*^ | 5.884 | .002 | 7.890 | 32.177 |
|  | Alpha-pinene10 | 21.936^*^ | 5.884 | .001 | 9.793 | 34.080 |
|  | Alpha-pinene5+Hypoxia | 4.863 | 5.884 | .417 | -7.281 | 17.006 |
| Based on estimated marginal means | |  |  |  |  |  |
| *. The mean difference is significant at the .05 level. | | |  |  |  |  |
| a. Adjustment for multiple comparisons: Least Significant Difference (equivalent to no adjustments). | | | | | |  |

| **Paired Samples Test** | | | | | | | | | | |
| --- | --- | --- | --- | --- | --- | --- | --- | --- | --- | --- |
| groups | | | Paired Differences | | | | | t | df | Sig. (2-tailed) |
|  |  |  | Mean | Std. Deviation | Std. Error Mean | 95% Confidence Interval of the Difference | |  |  |  |
|  |  |  |  |  |  | Lower | Upper |  |  |  |
| Control | Pair 1 | PND14 - PND54 | 2.47320 | 7.45497 | 3.33397 | -6.78337 | 11.72978 | .742 | 4 | .499 |
| Hypoxia | Pair 1 | PND14 - PND54 | 2.90119E1 | 5.98146 | 2.67499 | 21.58489 | 36.43881 | 10.846 | 4 | .000 |
| Alpha-pinene5 | Pair 1 | PND14 - PND54 | 6.96809 | 14.38066 | 6.43123 | -10.88786 | 24.82404 | 1.083 | 4 | .340 |
| Alpha-pinene10 | Pair 1 | PND14 - PND54 | 8.48121 | 9.56001 | 4.27537 | -3.38910 | 20.35153 | 1.984 | 4 | .118 |
| Alpha-pinene5+Hypoxia | Pair 1 | PND14 - PND54 | 7.19767 | 29.81417 | 13.33330 | -29.82151 | 44.21684 | .540 | 4 | .618 |
| Alpha-pinene10+Hypoxia | Pair 1 | PND14 - PND54 | 5.48035 | 2.41129 | 1.07836 | 2.48634 | 8.47436 | 5.082 | 4 | .007 |
