# Supplementary figures and images for: Alpha-pinene ameliorate behavioral deficit induced by early postnatal hypoxia in the rat: study the inflammatory mechanism
Source: Sci Rep. 2024 Mar 17;14:6416. doi: 10.1038/s41598-024-56756-1 (PMC10944845; doi:10.1038/s41598-024-56756-1)

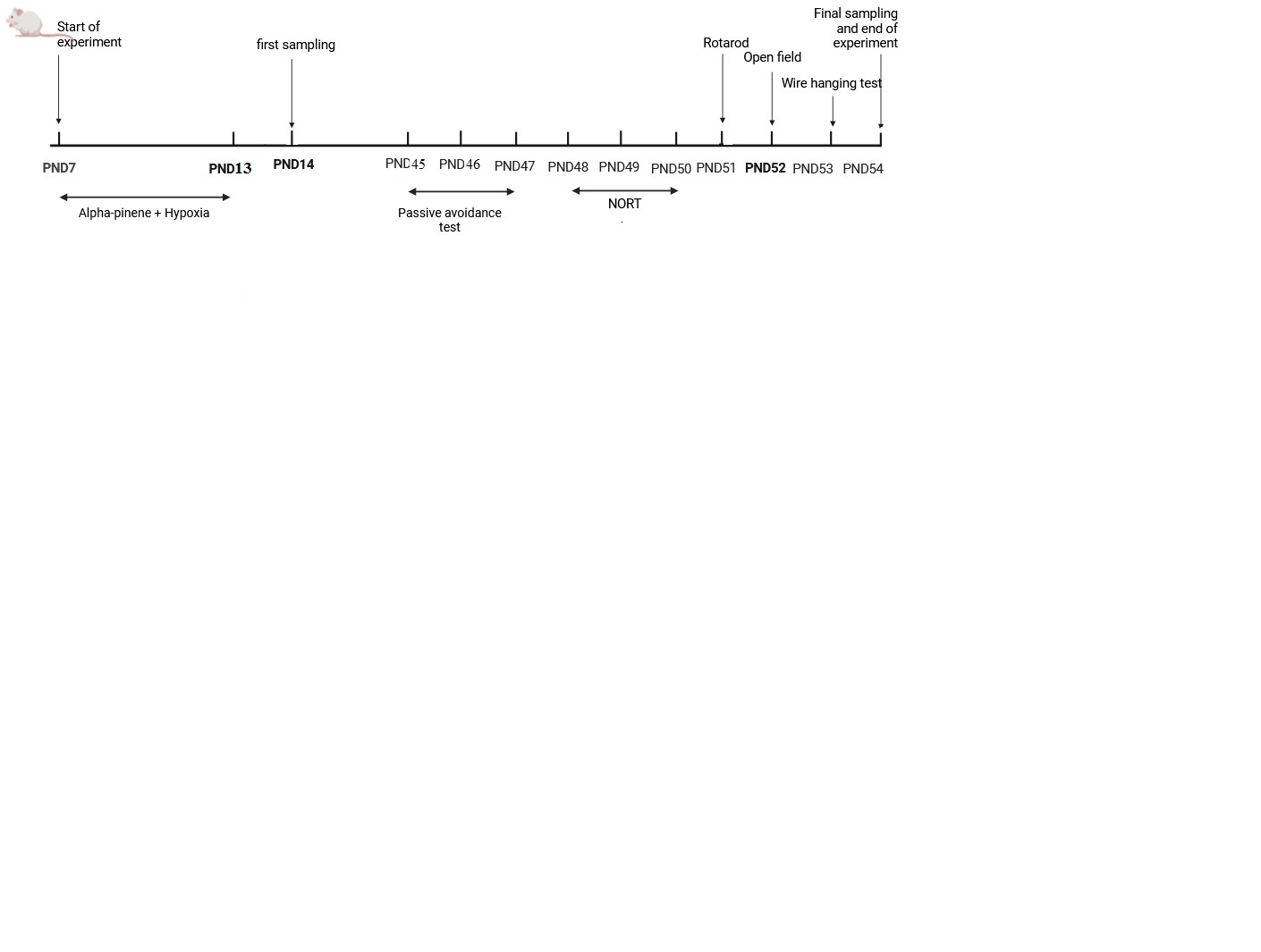

Supplement: Supplementary file 2 — Supplementary Information. [file 41598_2024_56756_MOESM2_ESM.jpg]
